# Molecular and clinical study on prevalence of feline herpesvirus type 1 and calicivirus in correlation with feline leukemia and immunodeficiency viruses

**Published:** 2014

**Authors:** Hamideh Najafi, Omid Madadgar, Shahram Jamshidi, Arash Ghalyanchi Langeroudi, Mahdieh Darzi Lemraski

**Affiliations:** 1 *Department of Microbiology and Immunology, Faculty of Veterinary Medicine, University of Tehran, Tehran, Iran; *; 2 *Department of Clinical Sciences, Faculty of Veterinary Medicine, University of Tehran, Tehran, Iran.*

**Keywords:** Corneal ulcers, Feline calicivirus, Feline herpesvirus type 1, Feline retroviruses, Respiratory disease

## Abstract

Upper respiratory tract diseases (URTD) are common clinical problem in cats worldwide. Feline calicivirus (FCV) and feline herpesvirus type 1 (FHV-1) are the main primary pathogens. Feline immunodeficiency virus (FIV) and Feline leukemia virus (FeLV) are also among the most common infectious diseases of cats which suppress the immunity. Oropharyngeal and conjunctival swabs and blood samples were taken from 16 cats with clinical signs of URTD and 26 clinically healthy cats. PCR and RT-PCR were used to detect FHV/FIV or FCV/FeLV infections, respectively. Feline calicivirus was detected in all cats with URTD and 87.00% and 93.00% of them were positive for FIV and FeLV, respectively. Feline herpesvirus rate of infection was 43.00% in sick cats. In clinically normal cats, prevalence rates of FCV and FHV were about 50.00%, but FIV and FeLV rates (42.00% and 65.00% respectively) were higher compared to other studies. Stomatitis was observed in 50.00% of cats with URTD. The main causative agent of corneal ulcers is FHV-1, but in 50.00% of cats with corneal ulcers, FCV was detected alone. It seems new variants of Caliciviruses are the main causative agents to attack uncommon tissues like cornea, although retroviral infections may be in the background of these various signs. The high retroviral prevalence may be due to existence of large population of stray cats. This is the first molecular study of FeLV and FCV in Iran and seems that FCV and FHV prevalence rates in FIV or FeLV infected cats is more than other non-infected ones.

## Introduction

Feline herpesvirus1 (FHV-1), a double-stranded DNA virus, member of the *Varicellovirus, *genus of the subfamily *Alphaherpesvirinae* combine with, feline calicivirus (FCV) that is a single-stranded positive-sense RNA virus, in the family *Caliciviridae*, genus *Vesivirus *are considered as the main agents involved in upper respiratory tract diseases (URTD) in cats.^1^^,^^2^ Despite the widespread use of vaccines against them in breeding catteries, infections with these viruses are still common, especially when cats are kept in groups.^3^

These viruses are responsible for acute illnesses and may cause recurrent or chronic lesions. Trigeminal ganglia are the site of FHV-1 latent infection and viral reactivation can occur during a stress period.^2^ The clinical signs in cats with FHV-1 and/or FCV infection include sneezing, an ocular and nasal discharge, conjunctivitis, dyspnea and coughing, while oral ulceration is common in cats with FCV.^4^ However, differentiation of diseases caused by FCV or FHV-1 on the base of clinical signs is almost impossible. ^5^

The FeLV and FIV are lymphotropic retroviruses that suppress the immune system of cats^1^^,^^6^ consequently cause a wide range of clinical signs.^7^^,^^8^ The FeLV replicates in the cells of the immune system producing a dramatic decrease in the populations of lymphocytes and granulocytes. FIV decrease both subsets of lymphocytes (CD4^+ ^and CD8^+^ T lymphocytes) and immune suppression occurs.^1^^,^^9^ In Iran, cats, (which are more often kept outdoors), are vaccinated routinely against rabies, feline pan-leukopenia, herpes-virus-1, and calicivirus, according to the American Association of Feline Practitioners vaccination guidelines.^10^ The risk of exposure to other feline pathogens, including FIV and FeLV is undetermined and no preventive programs are used for these diseases.^1^ However, it is important to determine the prevalence of FHV-1 and FCV and evaluation of their clinical signs, especially in relation with FIV and FeLV, to identify agents involved in URTD in Iran.

Despite routine vaccination, the numbers of cats with URTD referred to veterinary clinics are increasing. To define effective prophylactic and management programs, precise information on the prevalence of FeLV and FIV and their role in respiratory disease progress is required.

For better understanding the role of FIV and FeLV viruses in induction of FCV and FHV infections, the prevalence rates of these infections were investigated in healthy and diseased cats.

## Materials and Methods

**Fig. 1:**
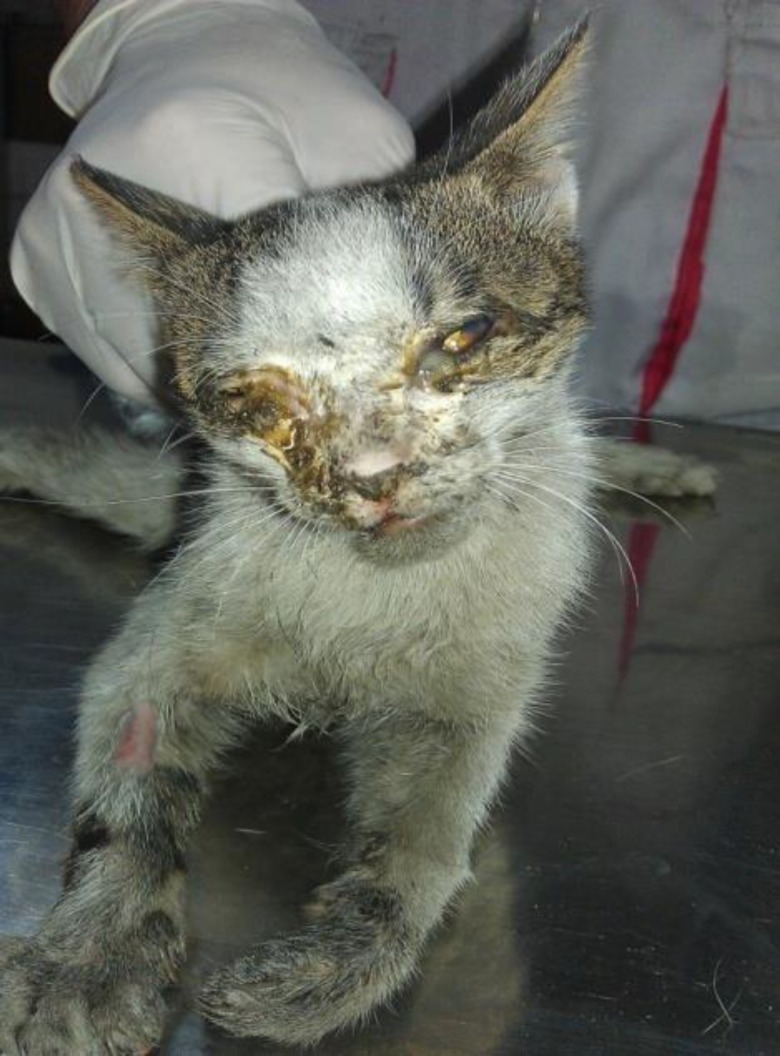
One of clinical cases with severe URTD in the present study. Nasal and ocular discharge with corneal ulcer was clearly observed in this case

**Animals. **A total of 42 non-vaccinated household cats from small animal hospital of University of Tehran were investigated in this study. Sixteen cats had clinical signs of URTD, including sneezing, coughing, nasal or ocular discharge, stomatitis, gingivitis and the other 26 were healthy and without any clinical signs of URTD. Figure 1 shows a cat with sever URTD symptoms which considered in this study.

**Samples. **For detection of FCV/FHV-1, oropharyngeal and conjunctival swabs were collected in tubes containing 1 mL PBS. After discarding swabs, tubes were centrifuged at 20,000 rpm for 10 min and the supernatant was discarded. The pellet obtained was washed three times with PBS and centrifuged to obtain a clean white pellet.

For detection of FIV/FeLV, a blood sample (0.5 to 1.5 mL) was collected from each cat by venipunctures containing EDTA. Tubes were centrifuged at 3,000 rpm for 10 min and buffy coat layers were collected.

**Nucleic acid extraction. **The nucleic acids were extracted from specimens (buffy coats and pellets) using the viral gene-spin kit (Intron Biotechnology, Seongnam, South Korea) according to the manufacturer’s instructions. Briefly, 300 µL of PBS containing virus samples, and 500 µL lysis buffer were mixed by vortex. After adding proteinase-k, samples were incubated at 55 ˚C for 10 min and centrifuged for 1 min at 13,000 rpm. A volume of 700 µL binding buffer was added and shake gently and 500 µL washing buffer-A was added to suspension and centrifuged for 1 min at 13,000 rpm and then, this step was repeated by washing buffer-B. Finally, 30 µL elution buffer was added and after centrifuging, extracted nucleic acid was collected.

**Primers. **Two pairs of oligonucleotide primers were used for the amplifying reaction. We used the previously designed primer sequences by Sykes *et al*.,^5^ for FHV-1 and the other primer which designed by Scansen *et al*.^11^ for FCV. HerpF (5'-GACGTGGTGAATTATCAGC-3') and HerpR (5'-CAACTAGATTTCCACCAGGA-3') amplify a 292 base pair (bp) region in thymidine kinase (TK) gene of FHV-1. CalcapF (5'-TTCGGCCTTTTGTGTTCC-3') and CalcapR (5'- TTGAGAATTGAACACATCAATAGATC-3') amplify a 126 bp region of the p30 gene of ORF1 of the FCV genome.

**Reverse transcription. **Reverse transcription were performed on ribonucleic acids (RNA) extracted from swab samples by 2-steps RT-PCR kit (Vivantis, Shah Alam, Malaysia). A volume of 8 µL RNA, 1 µL random hexamer primer (50 ng concentrations) and 1 µL dNTP mix (10 mM) were mixed and incubate in 65 ˚C for 5 min and then were placed on ice. Then 0.5 µL M-MuLV reverse transcriptase enzyme (100 unit) and 2.5 µL of 10X Buffer M-MulV and 7.5 µL nuclease-free water were added and placed in 42 ˚C for 60 min and then 85 ˚C for 10 min.

**Polymerase chain reaction. **Reverse transcriptase polymerase chain reaction (RT-PCR) was performed by method published by Scansen *et al*.,^11^ and PCR was performed according to the study of Sykes *et al*.^5^ for FCV and FHV-1 detection, respectively. Briefly, 2.5 µL of 10X PCR buffer, 0.75μl µL Mgcl_2_ (50 mM), 0.25 µL *Taq* DNA polymerase, 1 μlµL dNTP mix (10 mM) and 1 µL from each primers (10 mM) were added and then total volume reached to 22 µL with distilled water. Finally, 3 µL cDNA was added to it and were placed in thermocycler. For FHV-1, after an initial denaturation period of 5min at 95 ˚C, reactions were subjected to 40 cycles of 1 min at 91 ˚C, 1 min at 56 ˚C and 1min at 72 ˚C and final extension of 10 min at 72 ˚C. For RT-PCR amplification of FCV nucleic acid, we performed changes in thermocycler program which introduced by Scansen *et al*.^11^ Initial denaturation period was 5 min at 94 ˚C then, reactions were subjected to 40 cycles of 1 min at 94 ˚C, 1 min at 53 ˚C and 1min at 72 ˚C and final extension of 7 min at 72 ˚C. Each 15 µL of reaction products was electrophoresed through a 1.50% agarose gel and were stained with ethidium bromide. The appropriate molecular weight markers (100-bp DNA ladder; Sinaclon, Karaj, Iran) were used. The positive control included the extracted nucleic acid of the commercial strains of the vaccine (NOBIVAC, Cambridge, UK) and the negative control consisted of all the RT-PCR/PCR reagents except the nucleic acid; these were included in each reaction.

The FIV and FeLV reverse transcription and poly-merase chain reaction were performed by one-step PCR and one-step RT-PCR kit, respectively (Intron, Seoul, South Korea). A volume of 2 µL of extracted nucleic acid and 8 µL of DNase/RNase-free water were added to premix tube and after pipetting, tubes were placed in thermocycler. For FIV Initial denaturation period was 5 min at 94˚C then, reactions were subjected to 40 cycles of 30 sec at 94 ˚C, 30 seconds at 52 ˚C and 40 sec at 72 ˚C and final extension of 5 min at 72 ˚C. Electrophoresis and staining were performed as described above. Program used for RT-PCR amplification of FeLV nucleic acid is similar to program used for FIV amplification, but has a reverse transcription reaction time of 30 min at 95 ˚C at first and annealing temperature of 50 ˚C instead of 52 ˚C.

## Results

In cats with URTD, the prevalence was higher for FCV (100%) than for FHV-1 (43.00%) but in clinically normal cats the prevalence was about 50.00% for each virus. Some common clinical signs between both FCV and FHV infections were just seen in cats with FCV (Table 1). For example, sneezing and coughing never observed in FHV-1 positive cats. Also, prevalence rate of co-infection in both viruses was 30.00% totally (in sick and healthy animals), which just half of them showed clinical disease (Table 2). According to the results, rate of infection with these two viruses, especially FCV, in domestic cats were higher than other reports. Stomatitis was seen in 50.00% of cats with URTD. On our surprise, in 50.00% of cats with corneal ulcers, both FHV and FCV were detected, but in remaining 50.00%, we only found FCV though it has been shown that corneal ulcers are associated with FHV-1 infection (Table 1). Cats showing clinical signs of URTD were all less than 6 months of age. There was no association between FHV or FCV infections with gender and outdoor access of cats.

**Fig. 2:**
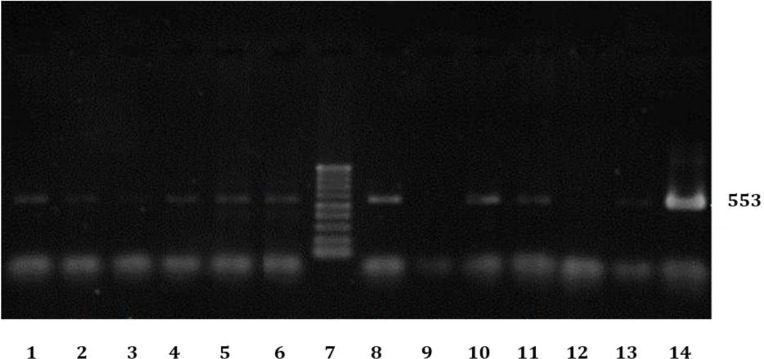
Electrophoresis results of some PCR products of FIV infected cats. No 7: Ladder (100bp DNA Ladder), 9: Negative control, 14: Positive control, 1-6 and 8 and 10-13 are results of some cats with URTD

**Fig. 3:**
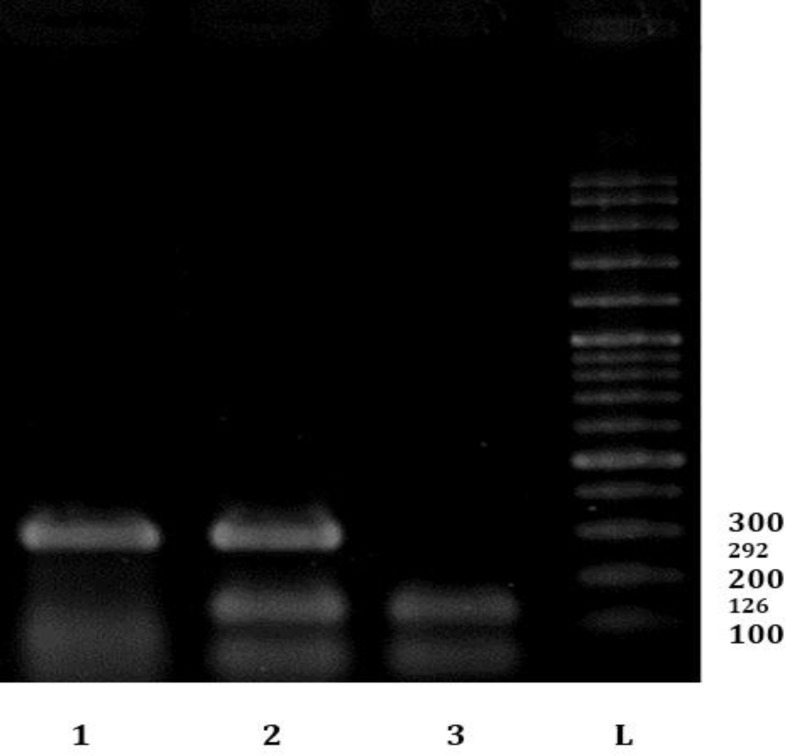
Electrophoresis results of some PCR products; two samples infected with FHV-1 and FCV and a vaccine which have both FCV and FHV-1 (Positive control, NOBIVAC, Cambridge, UK). L: Ladder (100 bp DNA ladder), 1: Positive sample for FHV-1, 2: Vaccine, 3: Positive sample for FCV

**Fig. 4:**
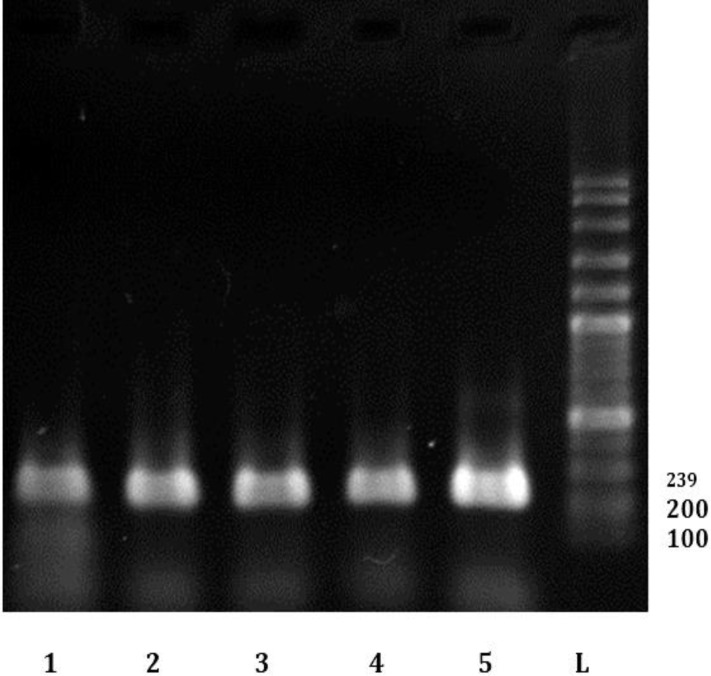
Electrophoresis results of some PCR products of FeLV infected cats L: Ladder (100BP dna Ladder). 5: Positive control. 1-4: Some positive sample for FeLV in this study

Also, prevalence rate of FIV and FeLV infections in cats with URTD (87.00% and 93.00%, respectively) were significantly higher than its rates in normal cats (42.00% and 65.00%, respectively). Male and outdoor cats were more infected than female and indoors (Tables 2 and 3). Interestingly, 43.00% of cats with URTD were positive for all viruses (FHV-1, FCV, FIV and FeLV). Figures 2, 3 and 4 show results of PCR (or RT-PCR) for FIV, FCV1, FHV and FeLV.

**Table 1:** Association between clinical signs in cats with URTD and infection with FIV, FeLV, FCV, FHV-1.

**Test result**	**Corneal ulcer (%)**	**Stomatitis (%)**	**Conjunctivitis (%)**	**Gingivitis (%)**	**Sneezing (%)**	**Coughing (%)**	**Lameness (%)**
**FHV** ^+^	71.00	28.00	42.00	14.00	0.00	0.00	12.00
**FCV** ^+^	68.00	50.00	43.00	31.00	31.00	31.00	12.00
**FIV** ^+^	57.00	50.00	42.00	35.00	28.00	21.00	14.00
**FeLV** ^+^	60.00	53.00	46.00	33.00	26.00	26.00	13.00

**Table 2:** Rates of co-infections of FCV, FHV-1, FIV and FeLV in healthy cats and cats with URTD

**Viruses**	**Healthy cats (%)**		**Cats with URTD (%)**
**FHV-1 and FCV**		23.00	43.00
**FIV and FeLV**		42.00	87.00
**FHV-1, FCV, FIV, FeLV**		11.00	43.00

**Table 3:** Prevalence of each virus and some parameters in cats of this study

**Groups**	**Viruses**	**Prevalence (%)**	**Male (%)**	**< 6 months (%)**	**Outdoor access (%)**
**Clinically healthy cats**	FHV-1	50.00	69.00	46.00	76.00
	FCV	46.00	75.00	50.00	83.00
	FIV	42.00	63.00	63.00	63.00
	FelV	65.00	70.00	63.00	70.00
**Cats with URTD**	FHV-1	43.00	42.00	100	71.00
	FCV	100.00	50.00	100	68.00
	FIV	87.00	50.00	100	71.00
	FeLV	93.00	53.00	100	73.00

## Discussion

Due to importance of feline respiratory diseases and their increasing prevalence especially when cats are kept together it is required to investigate the causative microorganisms and their potential relationship. In this study we determined the prevalence rates of FHV-1 and FCV, as the main pathogens of URTD. The prevalence rates of FIV and FeLV infections, which are associated with oral cavity diseases, and can cause a systemic immune-suppression and a wide range of diseases in domestic cats were also determined.^8^^,^^12^

It is very difficult to determine the causative agents of the symptoms of (FCV or FHV-1 infections) on the basis of clinical signs, thus accurate diagnosis requires microbiological assays. PCR, virus isolation and serological assays have been used previously. PCR-based assays offer rapid, sensitive and inexpensive diagnosis.^5^ In the present study, PCR and RT-PCR assays were used to detect FHV-1 and FCV infections using the PCR methods developed by Sykes *et al*. and Scansen *et al*., respectively. ^5^^,^^11^

In other studies, FCV and FHV-1 prevalence were 0 to 29.00% and 0 to 54.00% in healthy cats,^3^^,^^13^^,^^14^ and 20.0 to 53.00% and 10.0 to 34.00% in cats with URTD,^5^ which are less than the prevalence levels found in our study.

As shown in Table 3, prevalence of FCV (100%) in cats with URTD was higher than FHV (43.00%) that is in agree-ment with other reports.^2^ High prevalence of FCV may relate to high resistance of Calicivirus in environment. Also, chronically infected queens with FCV may constantly shed virus and consequently susceptible kittens can be infected.^1^^,^^3^

We did not find significant correlation between genders and outdoor access with FHV-1 or FCV infections and it seems that FCV and FHV-1 distribution is greatly depends on routes of virus shedding and the time when samples have been taken. For example, latent carriers of FHV-1 are not expected to shed virus at the exact time of sampling or many cats stop shedding FCV within a month though some become chronic carriers.^2^^,^^3^

While FHV-1 is known responsible for corneal ulcers,^1^^,^^13^ in the current study FHV-1 was not detected in half of cats having corneal ulcers. The absence of viral genome in our samples may be due to periodic shedding and carrier state of FHV-1. Also, it has been shown that FCV can undergo considerable antigenic and genetic variations resulting in generation of new variants of FCV with ability to damage cornea.^1^

Commercially available diagnostic kits for FeLV and FIV infections are based on the p27 antigen and antibodies against viral p24 capsid protein, respectively. However, a proportion of infected cats can be in period of time when they are negative for p27 antigen in their blood.^1^^,^^9^ Thus, we used RT-PCR assay for FeLV that has both a high sensitivity (92.00%) and specificity (99.00%) and is also able to detect a large number of cats with low FeLV proviral loads that could be negative by other conventional methods.^15^ The PCR assay were also used for FIV detection because it has priority to Western immunobloting and immunofluorescence assays and is highly sensitive and specific to detect FIV when used in experimentally infected cats.^15^

Interestingly, the overall prevalence rate of the FIV and FeLV infections found in both healthy cats and cats with URTD (Table 3) were markedly higher than the prevalence previously reported in Iran and other countries. The FeLV and FIV in healthy cats have been reported 0 to 2.00% and 6.5 to 7.50% in Australia, 2.90% and 9.80% in Japan, 3.60% and 3.20% in Germany, respectively and 64.00% (FIV) in Iran.^16^^-^^18^ Prevalence of FIV and FeLV are between 0 to 30.00% in cats worldwide.^1^

In Iran, previous studies using PCR and RT-PCR methods showed that the infection rate were 64.00% for FIV in Isfahan,^19^ and 4.40% (for FIV) and 2.20% (for FeLV) in Tehran provinces.^20^^,^^21^ In south eastern of Iran prevalence of FIV and FeLV were 19.20% and 14.20%, respectively,^10^ and in Ahvaz, the prevalence for FIV was 10.50%.^22^

Generally, the FIV and FeLV infections are declining in most parts of the world as a result of prevention and vaccination programs. The possible reasons for the higher rates of retroviruses found in our study might be due to lack of any preventive and vaccination program against FIV and FeLV and also, the presence of large population of stray cats and outdoor access of household cats with them that increase the risk of transmission of FIV and FeLV between cat population.

The greater prevalence rates observed in males are in agreement with other studies in USA, Iran^19^^,^^22^^,^^23^^,^^24^ and Japan where the prevalence of FIV in male pet cats was three times higher than females.^18^ The prevalence rates were also greater in cats with outdoor access. In a study in Japan, for FIV and FeLV infections, the sero-prevalence tended to be higher in outdoor.^18^

Because of role of retroviruses in immunosuppression, we studied co-infection of these viruses and FCV and/or FHV-1 to understand the effect of retroviral infections on severity of clinical signs caused by URTD pathogens. FIV was detected in 87.50% and FeLV in all cats showing stomatitis. Results suggests FCV and FIV together may cause stomatitis and FeLV and/or FCV infections in synergism with FIV, enhance the severity of oral cavity disease. Additionally, a study in western Canada showed oral diseases were significantly associated with FIV positivity.^25^ In 55.00% of cats with corneal ulcers, FHV-1 was not detected but all of them were positive for FIV and FeLV, expressing the systemic immunosuppressive effect of retroviruses that allow microbial invasion to unusual organs. These results are consistent with other reports. In Australia, the prevalence of FeLV was 3.50% in all cats, 1.40% in healthy cats and 6.90% in sick cats. The prevalence of antibodies to FIV was 10.40% in all cats, 4.90% in healthy cats and 16.70% in sick cats attended the veterinary hospital.^26^ Also, in North Carolina State, 123 cats were tested for FIV antibodies. More clinically ill cats had titers against FIV than did healthy cats (15.00% vs. 3.60%). Previous or current illnesses in these FIV positive cats included several clinical signs such as chronic respiratory tract disease.^27^ In another investigation in California, 226 cats were studied for FIV, FCV and FeLV prevalence. FIV-infected cats which were co-infected with either FCV or FCV and FeLV, had the highest prevalence of oral cavity infections and the most severe oral lesions.^12^

Surveillance for FIV was performed in Iran (Tehran) in 1998 for the first time using 105 household cats. Two seropositive cats (1.60%) were detected by enzyme-linked immunosorbent assay. Infected cats were sick and showed signs of upper respiratory infection and stomatitis.^28^ Our results, suggest that URTD is more observed in cats infected with FIV or FeLV, than cats are not infected by retroviruses.

In conclusion, it seems that FCV plays more important role than FHV-1 in URTD. Cats which are negative for FHV-1 should be sampled periodically, to prove that negative answer is not due to periodic shedding of FHV-1. Disinfection programs in catteries should be performed to control FCV. Totally, it seems FCV and FHV-1 infection rates are higher in FIV or FeLV infected cats than non-infected ones. Besides, new variants of FCV could have caused this high prevalence rate of infection despite mass vaccination in Tehran.
